# Rituximab in the treatment of refractory or relapsing eosinophilic granulomatosis with polyangiitis (Churg-Strauss syndrome)

**DOI:** 10.1186/ar4313

**Published:** 2013-09-24

**Authors:** Jens Thiel, Fabian Hässler, Ulrich Salzer, Reinhard E Voll, Nils Venhoff

**Affiliations:** 1 Department of Rheumatology and Clinical Immunology, University Medical Center Freiburg, Hugstetterstrasse 55, 79106 Freiburg, Germany; 2 Center for Chronic Immunodeficiency, University Medical Center Freiburg, Hugstetterstrasse 55, 79106 Freiburg, Germany; 3 University Hospital Freiburg, Hugstetterstrasse 55, 79106 Freiburg, Germany

## Abstract

**Introduction:**

Eosinophilic granulomatosis with polyangiitis (EGPA) is part of antineutrophil cytoplasmic antibodies (ANCAs)-associated vasculitides. In EGPA small-vessel vasculitis is associated with eosinophilia and asthma. About 40% of EGPA patients are ANCA-positive, suggesting a role for B cells in the pathogenesis of EGPA. B cell-depleting therapy with rituximab (RTX) can be effective in ANCA-positive EGPA, but very few patients have been published to date. The role of RTX in the treatment of ANCA-negative EGPA is unclear.

**Methods:**

We report a single-center cohort of patients with eosinophilic granulomatosis with polyangiitis. Of these patients, nine (six ANCA-positive, three ANCA-negative) had been treated with RTX for relapsing or refractory disease on standard immunosuppressive treatment. In a retrospective analysis, data on treatment response, frequency of relapses, adverse events, and peripheral B-cell reconstitution were evaluated. Furthermore, serum immunoglobulin concentrations, ANCA status, and peripheral B cell subpopulations were assessed after RTX treatment.

**Results:**

All patients had high disease activity before RTX treatment. At presentation 3 months after RTX therapy, all ANCA-positive and ANCA-negative patients had responded to RTX, with one patient being in complete remission, and eight patients being in partial remission. After a mean follow-up of 9 months, C-reactive protein concentrations had normalized, eosinophils had significantly decreased, and prednisone had been tapered in all patients. In all patients, RTX therapy was combined with a standard immunosuppressive therapy. Within the 9-month observation period, no relapse was recorded. Three patients were preemptively retreated with RTX, and during the median follow-up time of 3 years, no relapse occurred in these patients. During the follow-up of 13 patient-years, five minor but no major infections were recorded.

**Conclusions:**

In our analysis on nine patients with EGPA resistant to standard therapy, rituximab proved to be an efficient and safe treatment for ANCA-positive and ANCA-negative patients. Preemptive retreatment with RTX, combined with standard maintenance immunosuppressants, resulted in a sustained treatment response. Prospective, randomized trials evaluating the use of RTX in EGPA are warranted.

## Introduction

ANCA-associated vasculitides (AAVs) are a heterogeneous group of autoimmune diseases, sharing the feature of small-vessel vasculitis. The spectrum of AAV comprises granulomatosis with polyangiitis (GPA), microscopic polyangiitis (MPA), and eosinophilic granulomatosis with polyangiitis (EGPA), the later formerly known as Churg-Strauss syndrome (CSS). In EGPA small-vessel vasculitis is associated with eosinophilia and asthma [1]. The clinical manifestations commonly seen in patients presenting with EGPA range from upper airway and lung involvement to neurologic, cardiac, cutaneous, and renal manifestations [2-4]. The pathogenesis of the disease is incompletely understood, but an involvement of eosinophils and T lymphocytes has been demonstrated [5,6]. In EGPA patients, the peripheral T-cell compartment is skewed, and EGPA has been considered to be a Th2-mediated disease. Th2 cytokines like interleukin-5 (IL-5) function as growth factors for eosinophils [7] and eotaxin-3 has been identified as an eosinophil recruitment factor [8]. Targeting interleukin-5 with mepolizumab is promising for treatment of EGPA, but has a temporally limited effect. The conventional treatment of EGPA is based on glucocorticoids, which are combined with cyclophosphamide in patients with serious organ involvement. Depending on severity of the disease, immunosuppressants like methotrexate (MTX) or azathioprine (AZA) can also be used for remission induction and are often used along with glucocorticoids for maintenance therapy. To date, no clear disease-stage-specific therapy regimen exists for remission induction and maintenance therapy. The considerable rate of side effects related to the use of higher doses of glucocorticoids or cyclophosphamide, the high rate of relapses on standard therapy regimens, and the fact that some EGPA patients either do not respond to CYC therapy or relapse shortly after CYC treatment underline the need for alternative therapies [9]. Recent case reports suggest a favorable effect of the B cell-depleting agent rituximab (RTX) in EGPA [10-16]. The rationale for introducing a B cell-depleting therapy into the treatment of EGPA comes from the observation of myeloperoxidase (MPO)-specific ANCA in about 40% of EGPA patients [17], but the role of B cells in the pathogenesis of ANCA-negative EGPA is less clear. Furthermore, Th2 cells, by producing IL-4 and IL-13 may sustain the activation of not only eosinophils, but also B lymphocytes and promote B-cell class switching to IgE [6]. Eosinophilic granulocytes in turn maintain a vicious cycle of T-cell activation by secreting IL-25 [2]. Additionally, increased serum IgG4 concentrations have been described in EGPA [18]. RTX can induce remission in EGPA, but our knowledge on the role of RTX in EGPA is unfortunately based on a very limited number of case reports. Altogether, in studies reporting exclusively EGPA patients, fewer than 15 patients treated with RTX have been reported to date. We report nine EGPA patients from a single-center cohort that had been treated for relapsing or refractory disease on standard immunosuppressive treatment with RTX. We provide clinical data on relapse rate, peripheral B-cell reconstitution, and adverse events. Furthermore, we report on three EGPA patients that subsequently received RTX as part of a preemptive therapy strategy.

## Methods

### Selection of patients

Patients included in this study had a diagnosis of EGPA defined by the Lanham criteria [19], the American College of Rheumatology criteria [20], or the Chapel Hill Consensus criteria [21]. Furthermore, inclusion required RTX treatment for relapsing or refractory disease activity and a minimal follow-up after RTX infusion of 6 months. The study was approved by the ethics committee of the Albert-Ludwigs-University, Freiburg (file No. 191/11, 46/04). Written informed consent according to the Declaration of Helsinki was obtained from all patients.

### Study design

Patients included into this retrospective analysis were evaluated by a rheumatologist for diagnosis, sex, and age at application of first RTX infusion. Data analyses of the patients furthermore comprised laboratory values (for example, differential blood count, creatinine, C-reactive protein, creatine kinase, troponin T, myoglobin, lactate dehydrogenase, urine analysis), evaluation of disease extent, and organ involvement. Results of biopsies were included when available. Disease activity was measured by BVAS (version 3) [22]. The prognostic Five-Factor Score (FFS) was determined in all patients at first diagnosis of EGPA [23]. During follow-up after RTX application, patients were seen regularly every 3 to 6 months and evaluated by a rheumatologist.

### Definition of disease activity and treatment responses

Complete remission was defined as a BVAS of 0 for at least 3 months and a stable prednisone dose ≤7.5 mg prednisone per day. The persistent BVAS (pBVAS) rated disease status, when patients had some BVAS items that were not new or worsening but persistent (regressing or unchanged). A pBVAS >0 was defined to correspond to partial remission. Treatment response was defined as a 50% reduction of disease-activity score and absence of new manifestations attributable to ANCA vasculitis. Refractory disease was defined as unchanged or increased disease activity in acute EGPA after more than 4 weeks of treatment with standard therapy. Relapse was defined as the occurrence of ≥1 BVAS item caused by active AAV after having achieved remission.

Major relapses were defined as the recurrent or new onset of potentially life-threatening disease activity that could not be treated with an increase in glucocorticoids alone. All other relapses were defined as minor. Low-activity disease was defined as persistence of minor symptoms (for example, arthralgia or myalgia) [24].

### Analysis of immunoglobulins, ANCA, and peripheral B-cell numbers

RTX preceding immunosuppressive therapies, standard maintenance therapies after RTX, and concomitant use of glucocorticoids were assessed. Immunoglobulin (Ig) serum concentrations (normal range, IgG, 7 to 16 g/L; IgM, 0.4 to 2.3 g/L; IgA, 0.7 to 4 g/L; IgE, 10 to 100 IU/ml) were determined by nephelometry. The ANCA staining pattern (cytoplasmatic or perinuclear) was assessed by indirect immunofluorescence (Euroimmun, Medizinische Labordiagnostika AG, Luebeck, Germany). ANCA specificity for PR3 (Orgentec Diagnostika GmbH, Mainz, Germany) or MPO (Euroimmun, Medizinische Labordiagnostika AG, Luebeck, Germany) was measured by enzyme-linked immunosorbent assay (ELISA) and interpreted according to manufacturers’ reference, with values <10 IU/ml for PR3 and <20 IU/ml for MPO indicating a negative test. Peripheral B-cell counts were measured by using a whole-blood staining by flow cytometry by using a FACS Canto II (BD-Biosciences) and PE-Cy7-conjugated anti-CD19 (clone:J3.119; Beckman-Coulter). Results were expressed both as absolute cell counts and relative percentages with normal ranges, as previously described [25]. Depletion of B cells was defined as ≤0.01 × 10^9^/L or ≤0.5% of total peripheral lymphocytes. The start of peripheral B-lymphocyte repopulation was defined as B cells reaching >0.5% of total peripheral lymphocytes or >0.01 × 10^9^/L.

### Antibodies and flow-cytometric analysis of B-lymphocyte subsets in peripheral blood

PBMCs obtained from EDTA-anticoagulated blood by Ficoll density centrifugation were stained with the following fluorescent-labeled monoclonal antibodies: FITC-conjugated mouse-IgG1 (Beckman-Coulter), anti-CD27 (clone, M-T271; DakoCytomation), anti-kappa (clone, G20-193; BD-Biosciences), anti-CD10 (clone, ALB1; Beckman-Coulter), PE-conjugated anti-IgD (goat IgG F(ab)2; SouthernBiotech), anti-IgA (goat IgG F(ab)2; SouthernBiotech), anti-Lambda (clone, JDC-12; BD Biosciences) and anti-CD21 (clone, B-ly4; BD Biosciences), PE-Cy7-conjugated anti-CD19 (clone, J3.119; Beckman-Coulter); PerCP-conjugated anti-CD20 (clone, 2H7; Biolegend), APC-conjugated anti-CD45 (clone, 2D1; BD Biosciences), DyLight649-conjugated anti-IgM (goat IgG F(ab)2; Jackson ImmunoResearch Laboratories) and PacificBlue-conjugated anti-CD38 (clone, HIT2; EXBIO).

Immediately after isolation of cells, staining and flow-cytometric analysis were performed by using a FACS Canto II (BD Biosciences). After proper calibration by standard fluorescent beads, acquisition and analysis of 5,000 to 10,000 B cells were performed by gating on CD19^+^ events and lymphocyte characteristics by using both forward and sideward scatter, supported by the DIVA software (BD Biosciences). The following B-cell subsets were distinguished:

Total B cells, CD19^+^

Naïve B cells, CD19^+^IgD^high^IgM^+^CD27^-^

Transitional B cells, CD19^+^CD27^-^CD10^+^CD38^+^IgM^high^IgD^high^

Marginal zone B cells, CD19^+^IgD^+^IgM^high^CD27^+^

Switched memory B cells, CD19^+^IgD^-^IgM^-^CD27^+^

IgA B cells, CD19^+^IgA^+^

Plasmablasts, CD19^+^CD20^-^CD38^high^IgM^-/low^

CD21 low B cells, CD19^+^CD21^low^CD38^low^IgM^+^

Results were expressed both as absolute cell counts and relative percentages.

### Statistical analyses

Results were compared by Wilcoxon analysis by using SigmaStat software version 11.0 (Systat Software, Inc., SigmaStat for Windows). All statistical tests were two-tailed, and a *P* value <0.05 was considered statistically significant. When not otherwise indicated, median and interquartile range (median; IQR) are reported.

## Results

### Patients’ characteristics

The 160 patients with ANCA-associated vasculitides regularly attending the Department of Rheumatology at the University Medical Center for Vasculitides in Freiburg, Germany, were screened for this study. In total, 41 fulfilled the diagnostic criteria for EGPA. Of the 41 patients with a diagnosis of EGPA, nine patients had been treated with RTX for refractory disease activity (*n* = 8) or relapse (*n* = 1) on standard immunosuppressive therapy (Table 1). All nine patients (three women; six men), except for one, had a five-factor score of ≥1 at diagnosis. Mean age was 45 years (SEM, 5.5 years). All patients were of Caucasian origin and had systemic disease. Six patients were ANCA positive on indirect immunofluorescence screening, with a perinuclear fluorescence pattern in five patients and a cytoplasmic pattern in one patient. Myeloperoxidase (MPO) antibodies were present in five patients (Table 1). Median MPO titer at diagnosis was 108 U/ml (IQR, 64 to 132). Three patients were ANCA negative on indirect immunofluorescence screening and in PR3- and MPO-ELISA. Median concentration of C-reactive protein (CRP) before induction therapy was 69 mg/L (IQR, 43 to 119) (normal range below <3.2 mg/L), and median eosinophil count was 3,118/μl (31%) (IQR, 860 to 6,335). Median BVAS before induction therapy was 18 (IQR, 13 to 21). All patients had high disease activity with a baseline BVAS of ≥10 and pulmonary involvement with asthma in all, and nonfixed pulmonary infiltrates in seven (73%) of nine patients. ENT involvement was diagnosed in eight of nine patients. Neurologic manifestations were found in seven patients, all presenting with peripheral neuropathy, and two additionally having central nervous system involvement. Five patients had cardiac involvement with cardiomyopathy and/or pericarditis. Two patients reported ischemic cardiac pain, but results of coronary angiography were normal. Three patients had vasculitic skin lesions, and three patients had renal involvement (Table 1).

**Table 1:** Patients’ characteristics

**Number**	**Disease duration (months)**	**Organ involvement**	**FFS**	**ANCA IF ELISA**	**BVAS before induction**	**BVAS at RTX infusion**	**Organ involvement at RTX**	**Eosinophils/μl (%)**	**IgE IU/ml**	**Previous treatment**	**CYC (sum dose, g)**	**GC dose (mg)**	**Concomitant treatment after RTX**	**Total observation (months)**	**Additional RTX courses**
1	24	L, ENT, C	2	p	10	10	L↔, ENT↔, C↑	281 (6.9)	50	None	7.1	15	AZA	19	None
				MPO											
2	12	L, ENT, C, PNS	1	(p)	18	18	L↑, ENT↑, C↔, PNS ↔	8,677 (43)	869	AZA	9.8	40	AZA	6	None
				(-)											
3	66	L, ENT, PNS, CNS	1	p	14	14	L↓, ENT↔, PNS↑, CNS↑	663 (6.2)	269	MTX, AZA	(-)	10	AZA	6	None
				MPO											
4	5	L, ENT, C, PNS, S	2	(-)	28	16	L↔, ENT↔, C↓, PNS↑, S↓	1,057 (9.7)	1,030	None	10	25	AZA	32	1 more course at 6 months (preemptive)
				(-)											
5	16	L, ENT, C	1	(-)	19	19	L↑, ENT↔, C↔	4,235 (35)	76	MMF, AZA	9.7	20	AZA	34	3 more courses 6 monthly (preemptive)
				(-)											
6	184	L, ENT, PNS, S, K	1	p	19	13	L↓, ENT↑, PNS↑, S↑, K↓	3,542 (22)	17	CYC, AZA, MTX, LEF	8	7.5	MMF	36	5 more courses 6 monthly (preemptive)
				MPO											
7	35	L, ENT, C, S, PNS, CNS, K	1	(-)	36	23	L↑, ENT↓, C↑, S↓, PNS↔, CNS↓, K↓	8,434 (41)	528	CYC, MTX	12	40	AZA	13	None
				(-)											
8	7	L, PNS, K	1	p	13	9	L↔, PNS↑, K↓	2,700 (27)	332	AZA	9.9	15	AZA	6	None
				MPO											
9	8	L, ENT, PNS	0	c	13	10	L↑, ENT↑, PNS↑	3,536 (52)	206	MTX	(-)	15	MTX	6	None
				MPO											

The disease duration refers to the time from diagnosis to first RTX treatment. BVAS and organ involvement was evaluated before CYC induction and before the first RTX treatment. Eosinophil counts and IgE concentration were measured before induction therapy with CYC (*n* = 7), MTX (*n* = 1), or AZA (*n* = 1). Abbreviations for organ involvement: ear nose throat (ENT), cardiac (C), kidney (K), lung (L), peripheral nervous system (PNS), central nervous system (CNS), and skin (S). FFS, five-factor score; IF, immunofluorescence; ELISA, enzyme-linked immunosorbent assay; BVAS, Birmingham Vasculitis Activity Score. Arrows, organ involvement at the time of RTX treatment compared with initial manifestation before standard therapy: ↑, worsening of organ involvement; ↓, improvement of organ involvement; ↔ stable organ involvement.

### Treatment characteristics of EGPA patients before RTX therapy

**Figure 1:**
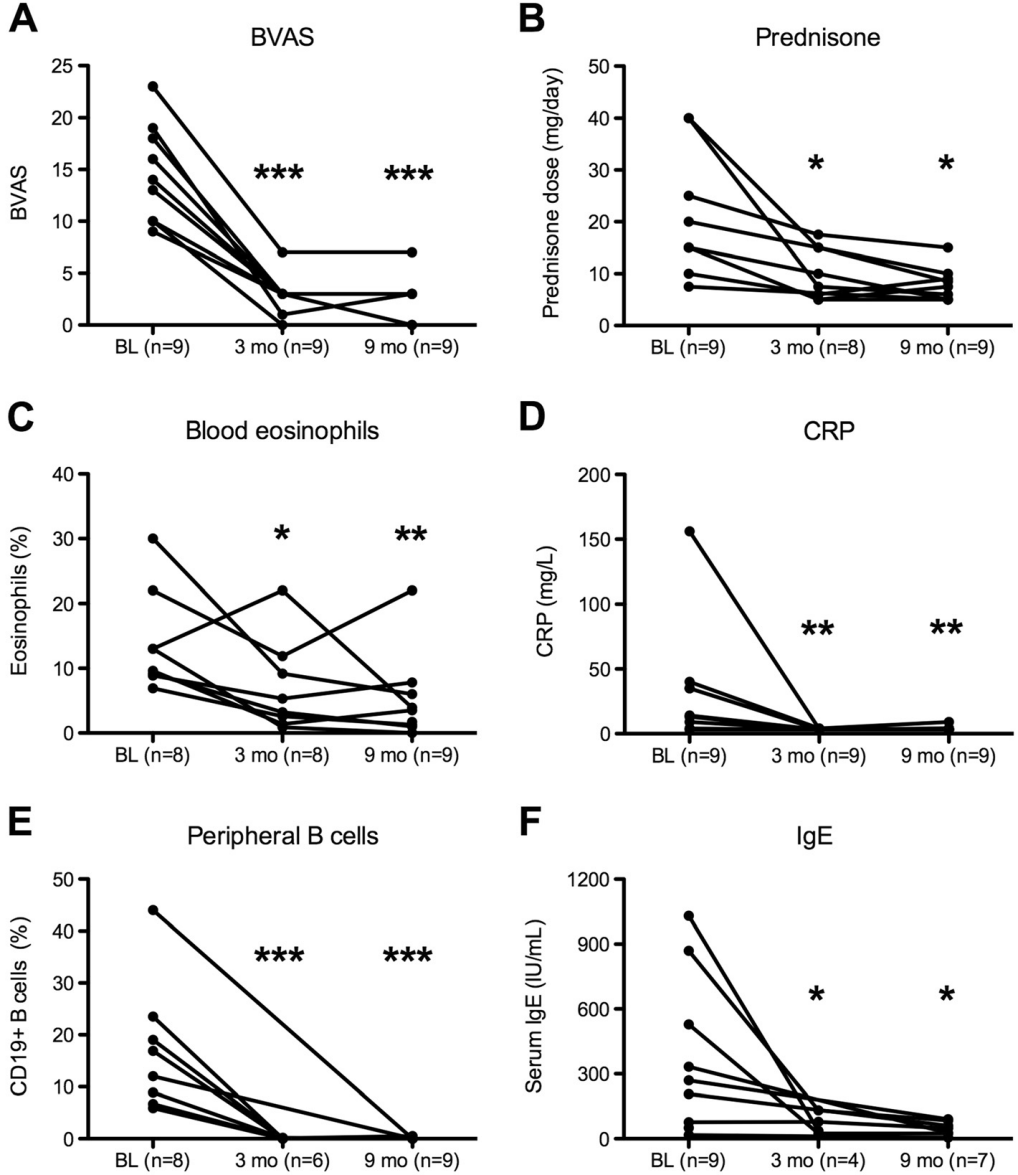
**Treatment response at a median follow-up of 3 months and 9 months after RTX treatment compared with baseline (BL).** Changes in BVAS **(A)**, daily prednisone dose **(B)**, eosinophils **(C)**, CRP concentrations **(D)**, B-lymphocyte percentages **(E)**, and IgE concentrations **(F)** are depicted. ***P < 0.001, **P < 0.01; *P < 0.05.

For induction therapy, seven patients were treated with intravenous cyclophosphamide (CYC) combined with glucocorticoids, and one patient each was treated with AZA (*n* = 1) or MTX (*n* = 1) together with glucocorticoids (Table 1). All patients were refractory to CYC therapy, and we found no statistically significant decrease in BVAS induced by CYC treatment (*P* = 0.286; MWU). The patients were therefore subsequently treated with RTX. In detail, after a median of three CYC boluses administered monthly (median dose of 1,000 mg per bolus), four patients had persistent severe cardiac involvement, five patients had persistently active or deteriorating peripheral neuropathy, three patients had increasing pulmonary infiltrates, and one patient had skin vasculitis. The median prednisone dose in these patients was 20 mg/day. Additionally, in two patients, ENT involvement had worsened, and in five patients, symptoms of asthma had increased. For persisting disease activity, RTX induction regimen consisting of two infusions of 1 g each, given 2 weeks apart, was administered. No infusion-related adverse events occurred. Median CYC sum dose before start of RTX was 7 g (IQR, 2.75 to 9.8). Median prednisone dose at the application of RTX was 15 mg/day (IQR, 14 to 29). In four patients (numbers 1, 4, 5, 8), CYC was continued as monthly infusions for a further 3 months after RTX treatment (median sum dose, 7.2; IQR, 5.4 to 7.3), and then replaced by an oral maintenance immunosuppressive therapy, whereas in four patients, RTX was directly followed by azathioprine (125 mg/day), and in one patient, MTX (15 mg/week) (Table 1). In all but one patient, BVAS before RTX application was still ≥10, and we found no statistically significant decrease compared with BVAS before CYC induction (*P* = 0.286) (Table 1). The median CRP concentration was 14 mg/L (IQR, 8 to 36), and median eosinophil count at diagnosis was 1,219/μl (11%) (IQR, 609 to 2,424) (Figure 1). Immunoglobulin concentrations for IgG, IgA and IgM before RTX therapy were within normal range. IgE concentration was increased to 305 IU/ml (IQR, 128 to 699). B-lymphocyte numbers before rituximab treatment were 193/μl (IQR, 68 to 338).

### Treatment response to RTX therapy

At presentation 3 months after RTX treatment, all patients (*n* = 9) had responded to RTX treatment, and median BVAS was 3 (IQR, 2 to 3), with a significant decrease compared with the median BVAS before RTX (*P* < 0.001) (Figure 1). The median decrease in BVAS from the last measurement before RTX was 10 (IQR, 7 to 12). One patient was in complete remission (number 1), and eight patients were in partial remission with persistent neuropathy (seven patients), and improved or stable ENT symptoms (two patients). In six patients, asthma symptoms improved after RTX treatment. Peripheral B cells were measured in six patients, and all analyzed patients had a complete B-cell depletion (median 0/μl; IQR, 0 to 1; and 0, IQR, 0 to 0.1). Median prednisone dose was 8.75 mg/day (IQR, 5.5 to 15; *P* = 0.036). Prednisone could be tapered by median 9 mg/day (IQR, 5 to 17.5). In six patients, ≤7.5 mg of prednisone was used as maintenance dose. Two patients had 10 mg prednisone per day because of residual asthmatic symptoms, and one patient had 15 mg/day because of recurrent asthma and increased numbers of eosinophils. CRP concentration had normalized (*P* = 0.004) in all patients, and eosinophil counts had decreased to 245/μl (4.3%; *P* = 0.05). IgE concentration normalized in all patients analyzed (*n* = 4) (Figure 1). In three patients, MPO titers were measured 3 months after RTX, and titers were within normal limits.

Mean follow-up after the first RTX course was 9 months (SEM, 1.7). Maintenance immunosuppressive therapy at follow-up analysis was AZA (150 mg/d; *n* = 7), MTX (15 mg/d; *n* = 1), MMF (1,000 mg; *n* = 1). Within the 9-month observation period, no relapse was recorded, and the median BVAS was 3 (IQR, 0 to 3) (Figure 1). At the last available time-point of observation (mean, 9 months) after the first RTX treatment, CRP concentrations were within normal limits in all patients, eosinophils had further decreased (median, 3.5%; *P* = 0.007), and IgE concentration was 48 IU/ml (*P* = 0.034) (Figure 1). Median prednisone dose was 7.5 mg/d. In no patient could beginning B-cell recovery be observed (Figure 1). Two of the nine patients (numbers 1 and 7) had a follow-up of more than 1 year after RTX application (13 months in one, and 19 months in another patient), and both neither relapsed, nor repopulated their peripheral B-cell compartment within this observation period. B-cell repopulation has been described to start between 6 and 9 months after B cell-depleting therapy. In four patients, the B-cell compartment was analyzed with high-sensitivity FACS analysis 6 months after RTX treatment.

None of these patients fulfilled the criteria of B-cell repopulation based on peripheral B-cell numbers and percentages. Therefore, a median of 850,000 events was recorded by FACS analysis to allow high-sensitivity analysis. In three of the four patients, events in the CD19^+^ gate were below 100, and these patients were excluded from further analyses. Only in one patient were we able to record more than 1,000 events in the CD19 gate. The B-cell subpopulations identified were mainly plasmablasts (CD19^+^CD20^-^CD38^high^IgM^-/low^) and switched memory B cells (CD19^+^IgD^-^IgM^-^CD27^+^). No transitional B cells were detected (data not shown). Three of the nine EGPA patients (numbers 4, 5, and 6) were subsequently retreated with RTX after a median of 6 months, as part of a preemptive therapy strategy. At retreatment, none of these patients had clinical signs of relapse; all had CRP concentrations within normal limits, and two patients had slightly increased but stable numbers of eosinophils. Both patients with increased numbers of eosinophils were ANCA-negative (patients 4 and 5), and had no PR3 or MPO antibodies before the initiation of RTX therapy. RTX courses (2 × 1 g given 2 weeks apart) were administered every 6 months, with two, four, and six courses in one patient each (Table 1). During the median follow-up time of 3 years in the patients on preemptive RTX treatment regimens, neither in the ANCA-positive nor in the ANCA-negative patients, were relapses observed. One patient (number 4) had a B-cell repopulation 22 months after the second RTX course.

### Immunoglobulin concentrations

All patients included in this study were evaluated for the serum immunoglobulin concentrations before RTX administration and after RTX treatment. Median immunoglobulin concentrations for IgG, IgA, and IgM before RTX therapy were within normal range (IgG, 13.2 g/L; IgA, 1.8 g/L; IgM, 1.2 g/L). After a median time of 24 months after RTX application, concentrations of immunoglobulins declined to IgG, 8.8 g/L (*P* = 0.005), IgA, 1.4 g/L (*P* = 0.122), and IgM, 0.4 g/L (*P* = 0.008). At this time, two patients (22%) had IgG concentrations below the lower limit of normal (7 g/L). None of the patients showed an increase in IgG concentrations during the observation time.

### Adverse events and infections

All patients included into this study were evaluated for adverse effects over a follow-up of 13 patient-years. RTX infusions were generally well tolerated, with no adverse events recorded that impeded the completion of the RTX infusion. No patient died during follow-up period of the study. No major infections required hospitalization, and five minor infections were recorded during the follow-up. All infections were related to the respiratory tract and treated with antibiotics. All patients that had airway infections had serum IgG concentrations <7 g/L. In one patient (number 5), a testicular seminoma was diagnosed 12 months after the first RTX course.

## Discussion

Eosinophilic granulomatosis with polyangiitis is part of the ANCA-associated vasculitides but differs in many aspects from other AAVs like GPA and MPA. Nevertheless, the treatment of EGPA is similar to treatment regimens used in GPA and MPA. A recent analysis on the long-term outcome of 118 patients with EGPA enrolled in two prospective trials reports a relapse rate of 41% at a mean of 26 months after treatment onset [9]. This underscores the need for treatment alternatives in EGPA patients. B cell-targeted therapy with rituximab has successfully been introduced into the therapeutic armamentarium of GPA and MPA, but to date, the role of RTX in EGPA therapy is unclear and based on few case reports comprising only a very limited number of patients [10-16]. When included in trials together with patients like other AAVs like GPA or MPA, EGPA patients were often not analyzed separately. We report on the successful treatment of EGPA patients with RTX. Most patients we report were resistant to conventional induction treatment with CYC, and some patients had relapsed on maintenance immunosuppressive therapy. All patients showed a rapid treatment response to rituximab, with decreasing BVAS, CRP concentration, and eosinophil counts, allowing tapering of prednisone. Recently, severe bronchospasm associated with rituximab for refractory EGPA was described in two patients [26], but no RTX infusion-related adverse events occurred in our patients that received prednisone and antihistaminics as comedication. At the evaluation 3 and 9 months after RTX infusion, the most frequent sequelae were persistent peripheral neuropathy and minor ENT symptoms that did not completely resolve. Eosinophil counts did not normalize completely in two ANCA-negative patients, but both nevertheless had clinically responded to RTX treatment. After RTX treatment, all ANCA-positive patients became ANCA negative. All patients stayed ANCA negative during the follow-up. B-cell counts were closely monitored in all patients after RTX treatment. In patients with rheumatoid arthritis treated with RTX, repopulation of the peripheral B-cell compartment starts 6 to 9 months after RTX therapy with an initial increase in transitional B cells. We analyzed four patients 6 months after RTX therapy with high-sensitivity FACS analysis, and found a persisting complete B-cell depletion in three patients. In one patient, few remaining switched memory B cells and plasmablasts but no transitional cells were found that would indicate a starting process of repopulation. Three of the nine EGPA patients were preemptively retreated with RTX. During the subsequent median follow-up time of 3 years, no relapse was observed. In these three patients, B cells were closely monitored during the follow-up time. Only one patient had a B-cell repopulation 22 months after the second RTX course. As our patients were initially treated with CYC and then with an oral maintenance immunosuppressive therapy after RTX, this may contribute to the delayed B-cell repopulation. Further studies assessing the repopulation kinetics of B cells after RTX treatment of EGPA patients are necessary.

During the follow-up of 13 patient-years, no major infections were recorded. In one patient, a testicular seminoma was diagnosed 12 months after the first RTX course, but most likely was unrelated to RTX treatment. Five minor infections related to the upper respiratory tract occurred during follow-up. As the patients in whom the minor infections were recorded had serum IgG concentrations slightly below the normal range, and we recently reported a decrease in serum immunoglobulin concentrations that may follow RTX treatment in AAV patients pretreated with CYC [27], we suggest that serum immunoglobulin concentrations should be measured regularly. A further evaluation addressing specific antibody production in EGPA patients after CYC/RTX treatment will help to assess the relevance of the decrease in serum immunoglobulins.

No opportunistic infections occurred in the patients we report. Furthermore, RTX treatment allowed a rapid tapering of daily prednisone, a major risk factor for infectious complications.

ANCA status separates two EGPA phenotypes that differ in organ manifestations, with renal and peripheral nervous system involvement being more common in ANCA-positive EGPA patients, whereas for ANCA-negative patients, an association with cardiac and gastrointestinal involvement, as well as pulmonary infiltrates, has been reported [17,28,29]. The presence of ANCA in EGPA pathogenetically differentiates a vasculitic form of the disease from an eosinophil-driven disease entity. The presence of ANCA suggests an involvement of B cells in disease pathogenesis and may provide a rationale for the use of a B cell-depleting therapeutic strategy. The rationale for the use of B-cell depletion in ANCA-negative EGPA patients seems to be less clear, but for all EGPA patients, a strong IgG4 immune response, irrespective of their ANCA status, has been described [18]. To our knowledge, very few ANCA-negative EGPA patients treated with rituximab have so far been described in the literature [11]. For GPA and MPA, it has been demonstrated that ANCA-negative patients can achieve remission with rituximab [30]. Furthermore, evidence of cross-talk between B cells and eosinophils and B cells has been described to be able to secrete eotaxin-1 [31], which in turn can recruit eosinophils [32]. Our study includes three ANCA-negative EGPA patients. All ANCA-negative patients responded to RTX therapy. Two ANCA-negative patients were treated with RTX as induction therapy, followed by RTX combined with azathioprine for maintenance therapy. As mentioned earlier, eosinophil counts decreased after RTX treatment but did not normalize completely in these two patients. Nevertheless, all ANCA-negative patients that initially had been resistant to a conventional immunosuppressive therapy, including cyclophosphamide, responded to RTX treatment. They achieved a partial remission only, with persistent symptoms of neuropathy. In the two patients re-treated with RTX preemptively, the treatment response was sustained during the whole follow-up period.

Our study has the limitation that the number of patients included and the follow-up time are limited. Therefore, we cannot draw valid conclusions from our data regarding long-term relapse-rate and infectious complications. Furthermore, seven of the nine patients reported were treated with CYC before RTX, and it cannot completely be excluded that a delayed effect of the CYC treatment contributed to the therapeutic response observed after RTX administration. But, as all patients reported had CYC-refractory disease after a median of three CYC i.v. boli, demonstrating a clear treatment failure of CYC, it does not seem plausible that the distinct treatment response observed after RTX administration is due to a protracted effect of the CYC treatment. Additionally, the patients included in the study were Caucasians from a single center. In our opinion, our study indicates that RTX is an effective therapy even in patients refractory to CYC, but because of the retrospective design and the limited number of patients included in our study we cannot deduce from our data whether a second immunosuppressant given concomitant with RTX is useful. Conversely, the strength of our study is that it is has no selection bias, as we report on a complete cohort of EGPA patients. In this way, our work differs from previous case reports. For case reports, it can never be ruled out that a selection bias exists, and predominantly patients with positive treatment responses are reported, whereas patients that did not respond to therapy are published less frequently.

## Conclusion

Our data on patients with EGPA resistant to standard therapy indicate that rituximab is an efficient and safe treatment for ANCA-positive and potentially also for ANCA-negative patients with EGPA. Preemptive retreatment with RTX combined with standard maintenance immunosuppressants resulted in a sustained treatment response. Prospective, randomized trials evaluating the use of RTX in EGPA are warranted.

## Abbreviations

AAV: ANCA-associated vasculitides; ANCA: Antineutrophil cytoplasmic antibody; AZA: Azathioprine; BVAS: Birmingham vasculitis activity score; CNS: Central nervous system; CRP: C-reactive protein; CSS: Churg-Strauss syndrome; CYC: Cyclophosphamide; EGPA: Eosinophilic granulomatosis with polyangiitis; ELISA: Enzyme-linked immunosorbent assay; ENT: Ear/nose/throat; FACS: Fluorescence-activated cell sorting; FFS: Five-factor score; GC: Glucocorticoid; GPA: Granulomatosis with polyangiitis; IF: Immunofluorescence; IL: Interleukin; IQR: Interquartile range; K: Kidney; L: Lung; MMF: Mycophenolate mofetil; MPA: Microscopic polyangiitis; MPO: Myeloperoxidase; MTX: Methotrexate; pBVAS: Persistent BVAS; PNS: Peripheral nervous system; PR3: Proteinase 3; RTX: Rituximab; S: Skin; Th: T-helper cell.

## Competing interests

The authors declare that they have no competing interests.

## Authors’ contributions

FH and US carried out the experiments, participated in the design of the study, and participated in writing the manuscript. JT, REV, and NV conceived the study, participated in its design and coordination, and analyzed the data. JT and NV wrote the manuscript. REV critically revised the manuscript. All authors read and approved the final manuscript.
